# Molecular Mechanisms of UV-Induced Apoptosis and Its Effects on Skin Residential Cells: The Implication in UV-Based Phototherapy

**DOI:** 10.3390/ijms14036414

**Published:** 2013-03-20

**Authors:** Chih-Hung Lee, Shi-Bei Wu, Chien-Hui Hong, Hsin-Su Yu, Yau-Huei Wei

**Affiliations:** 1 Department of Dermatology, Kaohsiung Municipal Hsiao-Kang Hospital, Kaohsiung 812, Taiwan; E-Mail: dermlee@gmail.com; 2 Department of Dermatology, Kaohsiung Medical University, Kaohsiung 807, Taiwan; E-Mail: dermyu@kmu.edu.tw; 3 Department of Dermatology, Kaohsiung Medical University Hospital, Kaohsiung 807, Taiwan; 4 Department of Biochemistry and Molecular Biology, National Yang-Ming University, Taipei 112, Taiwan; E-Mail: labboy700110@gmail.com; 5 Department of Dermatology, National Yang-Ming University, Taipei 112, Taiwan; E-Mail: zieben@gmail.com; 6 Department of Dermatology, Kaohsiung Veterans General Hospital, Kaohsiung City 813, Taiwan; 7 Department of Medicine, Mackay Medical College, New Taipei City 252, Taiwan

**Keywords:** UVR, apoptosis, oxidative stress, keratinocyte, langerhans cells, immunosuppression, phototherapy

## Abstract

The human skin is an integral system that acts as a physical and immunological barrier to outside pathogens, toxicants, and harmful irradiations. Environmental ultraviolet rays (UV) from the sun might potentially play a more active role in regulating several important biological responses in the context of global warming. UV rays first encounter the uppermost epidermal keratinocytes causing apoptosis. The molecular mechanisms of UV-induced apoptosis of keratinocytes include direct DNA damage (intrinsic), clustering of death receptors on the cell surface (extrinsic), and generation of ROS. When apoptotic keratinocytes are processed by adjacent immature Langerhans cells (LCs), the inappropriately activated Langerhans cells could result in immunosuppression. Furthermore, UV can deplete LCs in the epidermis and impair their migratory capacity, leading to their accumulation in the dermis. Intriguingly, receptor activator of NF-κB (RANK) activation of LCs by UV can induce the pro-survival and anti-apoptotic signals due to the upregulation of Bcl-xL, leading to the generation of regulatory T cells. Meanwhile, a physiological dosage of UV can also enhance melanocyte survival and melanogenesis. Analogous to its effect in keratinocytes, a therapeutic dosage of UV can induce cell cycle arrest, activate antioxidant and DNA repair enzymes, and induce apoptosis through translocation of the Bcl-2 family proteins in melanocytes to ensure genomic integrity and survival of melanocytes. Furthermore, UV can elicit the synthesis of vitamin D, an important molecule in calcium homeostasis of various types of skin cells contributing to DNA repair and immunomodulation. Taken together, the above-mentioned effects of UV on apoptosis and its related biological effects such as proliferation inhibition, melanin synthesis, and immunomodulations on skin residential cells have provided an integrated biochemical and molecular biological basis for phototherapy that has been widely used in the treatment of many dermatological diseases.

## 1. Introduction

The skin is the largest organ in the human body [1]. The skin covers the whole body surface and acts as a dynamic barrier to prevent water evaporation from the human body. It also prevents the entrance of noxious substances and pathogens into vital internal organs [2]. A network composed of delicate physical, chemical, and immunological barriers in the skin makes it a perfect organ to protect the integrity of the human body [3]. However, the integrity of skin barriers can be impaired by exogenous factors, including ultraviolet rays (UVR). In this review we discuss the effect of UVR on human skin with a focus on physiological and pathological apoptosis. Physiological apoptosis in the skin is reflected by the terminal differentiation of epidermal keratinocytes, which lose their nuclei when undergoing upward differentiation and manifest as gross scale shedding. Physiological apoptosis is important in governing normal skin turnover. The pathological apoptosis, on the other hand, may lead to benign proliferative inflammatory disease (such as psoriasis vulgaris) and neoplastic growth. We also review the application of UVR-based phototherapy in medical care focusing on apoptosis and its related biological effects in different skin residential cells.

## 2. Skin Physiology

**Figure 1:**
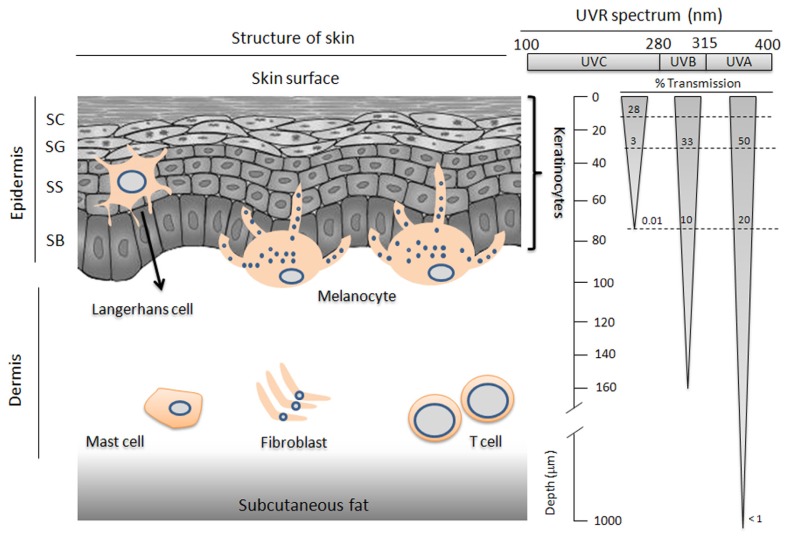
The structure of the skin and the penetration of solar ultraviolet rays (UVR) into the skin. The skin includes epidermis, dermis, and hypodermis. Melanocytes are localized in the basal layer and synthesize melanin. Langerhans cells (LC) are localized in the mid-epidermis and contribute to the presentation of antigens. While UVC carries the highest amount of energy, most of it is blocked by ozone layers. UVB carries an intermediate amount energy and it preferentially affects DNA in the cells. UVA carries the smallest amount of energy, but it penetrates deeply into the skin.

Anatomically, skin is divided into epidermis, dermis, and subcutaneous tissue, from the superficial to the deep tissues (Figure 1). Epidermis can be further divided into several layers, which include the basal layer, spinous layer, granular layer, and cornified layer, depending on the differentiation process of keratinocytes, the major cell type in the epidermis [4]. The keratinocytes in the cornified layer, the layer separating the outer environment from the inner host, lose their nuclei and their dead bodies are left along with extracellular compact molecules to contribute to the physical skin barrier. Thus, the terminal differentiation process in the cornified layer in this way represents the prototype of physiological apoptosis in the human epidermis [4]. In addition to keratinocytes, the main cells in the epidermis include melanocytes and Langerhans cells (LCs). Most of the melanocytes are distributed in basal layers, synthesizing and transferring melanin to adjacent keratinocytes, and contributing to skin color and photoprotection [5]. LCs are professional antigen presenting cells (APCs) in the epidermis of the skin, and their long dendritic structures comprise the first line immunological barriers [6]. Once activated by endogenous or exogenous antigens, they can migrate to skin draining lymph nodes and activate T cells [7]. T cells then traffic back to the skin to elicit an immune response against the antigens. Thus, LCs are pivotal directors of appropriate adoptive and adaptive immune responses. Under the epidermis the second layer of the skin, the dermis, is found, which is composed of many types of cells, including endothelial cells, lymphocytes, mast cells, and skin fibroblasts. This layer provides an important physical support and supplies nutrition to the epidermis [8]. The third skin layer, the subcutaneous fat, mostly contributes to thermal insulation [9].

## 3. The Biological Relevance of UVR to Skin

Depending on the wavelength, UVR (100–400 nm) can be divided into three parts—UVA (315–400 nm), UVB (280–315 nm), and UVC (100–280 nm) [10]. UVC has the shortest wavelength and the highest energy, although most of the solar UVC is blocked by the ozone layer. The maximum absorption spectrum of DNA (260 nm) lies in the UVC spectrum. Thus, UVC is most harmful to genetic integrity, but it seldom reaches human skin due to its absorption by ozone layers. UVA has the longest wavelength with the lowest energy and can penetrate deeply into the dermis and cause aging effects [11]. UVB, which has a wavelength spectrum in between UVC and UVA, can cause redness of the skin and contribute to most of the UVR entering the dermis. Since UVB is only partially blocked by clouds or fog, UVB radiation is considered as the main cause of sunburn and skin cancers [12]. In fact, both UVB and UVA radiation contribute to freckling, skin wrinkling and the development of skin cancers [13,14]. Since the skin is composed of different layers of varying depths with different physical and chemical properties, UVR exerts different biological effects on different kinds of cells in the skin (Figure 1).

## 4. UV-Induced Apoptosis of Keratinocytes

**Figure 2:**
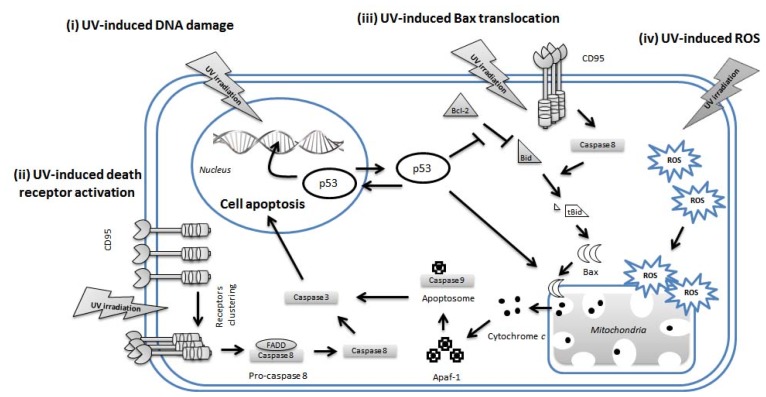
Mechanisms of UV-induced apoptosis. (i) UV induces DNA damages followed by p53 activation and leakage of cytochrome c from mitochondria; (ii) UV can also activate and cluster several cell membrane death receptors (extrinsic pathway), leading to activation of caspase cascade and apoptosis; In addition, (iii) UV-induced death receptor activation can further promote the Bax translocation to mitochondria, leading to the release of cytochrome c. Moreover, (iv) UV can induce overproduction of reaactove oxygen species (ROS), not only damaging several crucial structural and functional proteins and DNA, but also exerting the release of cytochrome c from impaired mitochondria.

DNA is the best-known target of UVB. UVB leads to two main photochemical reactions in DNA, including cyclobutane pyrimidine dimers (CPDs) and (6–4) pyrimidine-pyrimidone photoproducts [(6–4)PPs] [15,16]. Most photochemical products are caused by C→T and CC→TT mutations at dipyrimidine sequences in DNA [17]. Among these, CPDs are considered more mutagenic because of their abundance, slow repair and distinct mutagenicity [18]. These photolesions contribute to the high proportion of *p53* mutations in squamous cell carcinomas [19]. It has been reported that T-T lesions may help generate N-*Ras* mutations in murine squamous cell carcinomas [20] and in human melanomas [21]. If left unrepaired, the damaged DNA, as reflected by an increase in 8-hydroxy-2′-deoxyguanosine (8-OHdG) [15], may result in errors in DNA synthesis and genomic mutations, which may contribute to carcinogenesis in the context of active cell proliferation. A prototype of UV-induced apoptosis of keratinocytes is the formation of epidermal sunburn cells that are destined to apoptosis after sun exposure [22]. Thanks to the elimination by apoptosis of cells harboring genetic mutations, the host is able to prevent further incorporation of damaged and/or mutated genes in subsequent clonal cell expansion. The apoptotic effects of UV may depend on the dose and type of irradiated cells, which includes not only the p53-dependent apoptotic pathway but also the death receptor-dependent and mitochondrial dysfunction-mediated apoptotic pathways (Figure 2). Current understanding holds that p53 mediates mitochondria-dependent apoptosis through direct interaction with the mitochondrion itself and/or with members of the BCL-2 family of apoptosis-regulating proteins [23]. Recently, a mouse study showed the number of sunburn cells was decreased and DNA fragmentation was reduced in proportion to the UVA/UVB ratio [24]. Furthermore, the apoptosis induced by UVB and UVC in human HaCaT keratinocytes involves intrinsic and extrinsic programs [25]. UVA-induced CPD are formed via a direct photochemical mechanism without mediation of a cellular photosensitizer [26]. Exposure of cells to pure UVA radiation generates thymine cyclobutane dimers that are slightly less efficiently repaired than CPDs produced upon UVB irradiation [27]. In addition, UVA-induced DNA double-strand breaks can be generated from the repair of clustered oxidative DNA damages [28].

### 4.1. p53 in UVR-Induced Apoptosis of Keratinocytes

As a response to DNA damage, p53 and its downstream targets, p21 and Gadd45 are activated in the affected cells [29]. The p53 directly interacts with nucleotide excision repair (NER)-associated regulatory proteins. The mutations in the NER machinery can cause xeroderma pigmentosum (XP), an autosomal recessive disease with impaired DNA repair after UV radiation and early development of skin cancers [30]. Several studies have demonstrated that DNA repair is impaired in the absence of functional p53 [31]. Compared with the wild-type mice, knockout mice lacking the p53 protein show a reduction of sunburn cells in the epidermis following UVB irradiation [32]. Mutated p53 with defective function is commonly present in non-melanoma skin cancers and actinic keratosis, a premalignant lesion that may give rise to invasive squamous cell carcinoma [33]. On the other hand, basal keratinocytes also exhibit p53-independent apoptosis (described below) following UV radiation. However, upon induction of differentiation to committed progenitor cells, the apoptosis is dependent on p53-related signaling pathway. Although apoptosis is p53-independent in basal keratinocytes, DNA repair is p53-dependent in other cell types of skin tissues. Thus, p53 acts as an important regulator of DNA repair but it is not involved in the apoptosis of basal keratinocytes.

### 4.2. Extrinsic Pathways in UV-Induced Apoptosis

CD95 (Fas/APO-1), a member in the tumor necrosis factor (TNF) family receptors, is characterized by similar cysteine-rich extracellular domains and a homologous cytoplasmic sequence named the “death domain”. UVR induce multimerization of CD95, resulting in its binding to the adaptor protein Fas-associated protein with death domain (FADD), followed by commitment activation of caspase cascade from Caspase 8 to Caspase 3 [34]. In transformed HaCaT cells, one of the commonly used human keratinocytes, this extrinsic pathway appears to be important in apoptosis since HaCaT cells lack a functional p53 [35]. However, apoptosis cannot be totally attributed to this extrinsic pathway because a neutralizing anti-CD95 antibody blocks CD95L-induced apoptosis but fails to prevent UV-induced apoptosis [35]. On the other hand, tumor necrosis factor receptor (TNFR) is clustered and internalized in keratinocytes after UVB irradiation. This process is coupled with the recruitment of TNFR1-associated death protein and TNFR-associated factor-2 (TRAF-2) in the human or murine keratinocyte cell lines, respectively. There appears to be crosstalk between TNFR and FADD-induced apoptotic pathways [36]. In addition, the third receptor families influenced by UVR are the TRAIL receptors [37]. TRAIL receptors include two receptors that relay death signals (TRAIL-R1, -R2) and two receptors, which serve as decoy receptors due to their competitive binding to halt apoptosis. This balance might be altered by UVB irradiation, which at lower fluences might induce TRAIL-mediated apoptosis by inhibition of binding with decoy receptors [38].

### 4.3. Intrinsic Pathways in UV-Induced Apoptosis

The intrinsic pathway involved in UVR-induced apoptosis results from DNA damage and cytochrome *c* release from mitochondria (Figure 2) [39,40]. Permeation of the mitochondrial outer membrane [39,40] and leakage of cytochrome *c* into the cytosol triggers a caspase cascade. Once released, cytochrome *c* and the apoptotic protease activating factor-1 (Apaf-1) together form the apoptosome, a protein complex that recruits and activates Caspase 9 [41]. The balance of pro-apoptotic (Bax, Bak and Bid) and anti-apoptotic (Bcl-2 and Bcl-x) members of the Bcl-2 protein family determine the initiation or the inhibition of apoptosis [39]. Bcl-2 inhibits Caspase 3 and Caspase 8 activation, while Bcl-x partially inhibits cytochrome c release [42]. One of our previous studies showed that, in primary keratinocytes, UVB induces keratinocyte apoptosis via suppression of Bcl-2 expression (intrinsic) and activation of Caspase 8 (extrinsic) [43]. A combined use of UVB irradiation and arsenic treatments has been found to result in the anti-proliferative and pro-apoptotic effects by activation of Caspase 8 and 9 in keratinocytes [44,45]. It also has been reported that p53 can interact with the mitochondria-mediated pathway and Bcl-2 and Bcl-xL proteins to regulate apoptosis [46,47]. On the other hand, the epidermis contains several antioxidant enzymes including superoxide dismutase, glutathione peroxidase and catalase, which can remove ROS from the skin [48] and are depleted by prolonged exposure to UV [49]. Free radical scavengers, such as vitamins C and E, carotenoids and glutathione are also localized on the skin to prevent the damaging effects of ROS [50]. It has been documented that large-scale deletions of mitochondrial DNA (mtDNA) are present in sun-exposed skin tissues [51,52]. In one of our previous studies, we showed that a high proportion of mtDNA deletion rendered human cells more susceptible to UV-induced apoptosis through enhanced release of cytochrome c [53]. We demonstrated that human skin fibroblasts harboring pathogenic mutations of mtDNA were more susceptible to apoptosis triggered by UV irradiation or oxidative stress. This may provide a regulatory mechanism for the skin to exert quality control of mitochondria and to prevent further increase of oxidative damage or associated pathological changes under oxidative stress. Taken together, the results from the other investigators and our laboratory indicate that oxidative stress and damage elicited by mtDNA mutations not only lead to mitochondrial dysfunction but also increase the susceptibility of affected skin tissue cells to apoptosis upon UV irradiation [54–56].

## 5. The Modulation of Immune Responses by UVR through Apoptosis

In the epidermis, *trans*-urocanic acid (UCA) is present at high concentrations in the *stratum corneum* and absorbs UVB at short wavelength to attenuate the efficiency of light penetration into the skin [57]. UVR isomerizes *trans*-UCA to *cis*-UCA, which has potent immunosuppressive properties [58]. The *cis*-UCA induced by UV radiation stimulates the production of reactive oxygen species in keratinocytes, which leads to oxidative DNA damage and downstream immunosuppression [59]. Others suggest that *cis*-UCA is immunosuppressive in that it modulates the production of immune mediators from keratinocytes, nerves and mast cells [60]. In addition to the UCA chromophore, other chromophores in the skin include DNA and lipids in keratinocytes and APCs as well as tryptophan in skin cells. Since UV can induce apoptosis of keratinocytes, the apoptotic body is a potent inducer for immature LCs [61]. UVR can induce the depletion of LCs in the epidermis and impair the migratory capacity of LCs, leading to their accumulation in the dermis [62]. UV-induced DNA breaks in LCs could induce suppressive cytokines such as IL-10 and inhibit CHS responses [63]. In contrast, overexpression of DNA repair enzymes, such as T4 endonuclease V or photolyase, can efficiently repair UV-induced pyrimidine dimers and inhibit the local immunosuppression of cutaneous APCs and regulatory T cells (Treg) [64]. Actually, LCs are critical to the generation of Treg cells [65]. The defective LCs and Treg cells may mediate UV-induced suppression of contact hypersensitivity (CHS) [66]. Loser *et al.* demonstrated that the capacity of LCs to induce Treg cells is dependent on the activation of receptor activator of NF-κB (RANK) on the epidermal LCs through its ligand RANKL, also known as CD254, OPGL and TRANCE on keratinocytes [65]. A well-characterized consequence of RANK activation on LCs is an anti-apoptotic signal caused by the upregulation of Bcl-xL [67–69]. This mechanism may protect UV-damaged LCs from apoptosis until they reach the skin draining lymph nodes. These UV-exposed LCs have a high level of RANK, which can potently induce activation of Treg cells *in vitro*[65]. In addition, LCs, after escaping from UV-induced apoptosis, have a decreased capacity to prime naive CD8^+^ T cells, inducing immunological tolerance [70]. Together these findings show that LCs play an important role in generating antigen specific Treg cells that mediate UV-induced tolerance. In T cells, UVB can eliminate the mRNA of IL-12 and decrease interferon-gamma (IFN-) producing T-cells by over 50% [71,72]. Furthermore, IL-4 has been found to be increased by over 80% during UVB treatment of patients with psoriasis [73]. These results suggest that UVB is able to restore the proinflammatory-to-regulatory cytokine imbalance in inflammatory cutaneous disorders. The immunomodulatory effects of UV serve the basis of UV-based phototherapy. In fact, LCs derived from mice deficient in the pro-apoptotic Bid (BH3-interacting death domain protein) gene resist apoptosis and can induce amplified contact hypersensitivity reactions. Bid activation is a critical upstream mediator in UV-induced keratinocyte and LC apoptosis and its absence abrogates UV-induced immune tolerance [74].

## 6. UV Induces Melanogenesis and Apoptosis in Melanocytes Differentially Based on Wavelength and Dose

**Figure 3:**
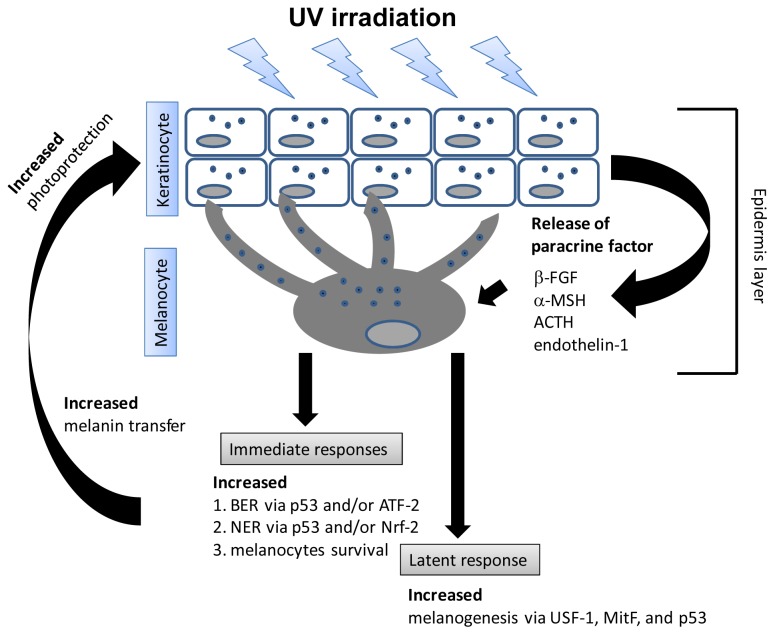
Physiological UVR dose induces activation of the transcription factors USF-1, Mitf, ATF-2, Nrf-2 and p53, and inhibition of NFkB. Similar as in keratinocytes, UV induces cell cycle arrest, activation of antioxidant and DNA repair pathways, and regulation of apoptotic pathways, to ensure melanocyte genomic integrity and survival in melanocytes.

Physiologically, UVR is known to induce synthesis of melanin in the melanocytes and melanin is important in the protection of harmful effects of UV (Figure 3) [75]. Several studies have reported that exposure of the skin to UV results in increased synthesis of paracrine factors, such as ACTH, endothelin-1, β-FGF, and α-MSH, which play an important role in mediating the UV response of human melanocytes [76]. For example, α-MSH is known to reduce the generation of UV-induced DNA photoproducts by enhancing nucleotide excision repair (NER) and to diminish the induction of oxidative DNA injury through elimination of ROS [77,78]. In addition to the induction of paracrine factors, UV can induce the activation of the transcription factors USF-1, Mitf, ATF-2, Nrf-2 and p53, and inhibition of NFκB [76]. The acute effect of UV includes execution of NER and promotion of melanocyte survival. The dynamics of melanogenesis induced by repeated exposures depends on UV dose, dose interval and emission spectrum with UVA generally being stronger than UVB to induce pigmentations [79]. There is also evidence that increasing the UV dose above a certain level of cumulative exposure does not significantly increase the level of UV-induced pigmentation [80]. Similar to that seen in keratinocytes, UV induces cell cycle arrest, activation of antioxidant and DNA repair enzymes, and regulation of apoptotic pathways in melanocytes, to ensure genomic integrity and survival of melanocytes [76]. These regulatory processes enhance melanogenesis to confer appropriate photoprotection of the epidermis against UV-induced damage (Figure 3). Therefore, unraveling the mechanisms by which the stress response of melanocytes to UV and more specifically, the regulation of DNA repair pathways, in melanocytes might lead to strategies to prevent malignant melanoma, a rapidly-fatal malignancy derived from melanocytes [76].

## 7. UV and Vitamin D Synthesis in Skin: Immunological Modulations

Despite the notorious effects of UV, it contributes to the synthesis of vitamin D, an important molecule in calcium homeostasis. Vitamin D is best synthesized by the skin following UVB exposure [81] and this synthesis may vary among individuals with different skin types [82]. Deficiency of vitamin D leads to osteoporosis and fractures and it is associated with cancers and autoimmune diseases such as rheumatoid arthritis [83]. The most biologically active vitamin D metabolite is 1,25-dihydroxyvitamin D3 [1,25(OH)_2_D3], which is synthesized locally in the skin and systemically after skin exposure to sunlight [84]. Rates of thymine-dimer repair and UV-induced apoptosis in the epidermis of vitamin D receptor (VDR) knockout mice have been found to be significantly lower than those in the epidermis of wild type mice [85]. Furthermore, UV-induced epidermal thickening has also been found to be attenuated in VDR^(−/−)^ skin, indicating that VDR plays a critical role in the repair and removal of severely damaged keratinocytes by UV exposure [85]. Apart from its active role in DNA repair, vitamin D also modulates the immune response after UV irradiation. Immune cells such as macrophages and dendritic cells (DCs) also can synthesize 1,25(OH)_2_D3. Intriguingly, local 1,25(OH)_2_D3 synthesis activates innate immune responses, but suppresses adaptive immune responses [86]. Cell differentiation reduces VDR expressions in macrophages and DCs, preventing mature immune cells from responding to vitamin D and facilitating a normal adaptive immune response [60]. Activation of Toll-like receptor 1 (TLR1) and TLR2 by tuberculosis enhances 1α-hydroxylase expression, the enzyme mediates the synthesis of 1,25(OH)_2_D3, production of antimicrobial peptide cathelicidin [87], and induction of tolerogenic DCs and T cells [60,84]. On the other hand, many cell types outside of the skeletal system, including various cells in the skin, also express the vitamin D receptor. Those cell types convert circulating 25-hydroxyvitamin D to 1,25-dihydroxyvitamin D for local use [81]. This metabolite exerts potent effects on cell differentiation, proliferation, and immune regulation. It is theorized that through these mechanisms vitamin D (as induced by UVR) and its analogues can be used to treat psoriasis and other hyperproliferative or inflammatory skin diseases. For example, UV radiation and topical 1,25(OH)_2_D3 can also activate dermal mast cells [88,89], which are important to determine the extent of UV immunomodulation [89,90].

## 8. Application of Phototherapy: UV-Induced Apoptosis and Biological Consequences

The first recorded UV therapy was performed by Dr. Niels Finsen, the 1903 Nobel Laureate in Physiology or Medicine, who demonstrated that UV has a positive effect on lupus vulgaris, a form of skin tuberculosis [91]. Although few theories exist how Finsen UV therapy worked against lupus vulgaris, Wulf’s group thought that Finsen used UVA radiation and that it acted through photosensitization and ROS production by porphyrins in the bacteria [92]. Martineau *et al*. suggested that the positive effect came from vitamin D synthesized by UVB, which was used to treat tuberculosis in the pre-antibiotic era [93]. The involvement of UV-induced apoptosis in this therapy is unclear, though photosensitization and oxidative injury may play a role. Table 1 lists several clinical techniques related to the phototherapy widely applied in medicine. Visible light in the blue-green range (430–490 nm) has been used as a standard treatment of neonatal jaundice, for example. The tissue bilirubin, possessing the heme group, absorbs the light in this spectrum and the metabolites become more lipophilic than the mother compound and are more readily excreted [92]. Although the mechanism is not related to apoptosis, the blue lamp has been shown to form more photo-oxidation products and cause more severe cellular damage and apoptosis in the presence of bilirubin as compared to the green lamp [93].

Currently, irradiations with broadband UVB (290–320 nm), narrowband UVB (311–313 nm), 308 nm excimer laser, UVA 1 (340–400 nm), UVA with psoralen (PUVA), and extracorporeal photochemotherapy (photopheresis) are in use [105]. Electromagnetic waves (EMW) in different wave lengths have various effects in the skin that occur as a result of photoselective thermolysis, evaporation, immunosuppression, abnormal DNA repair, apoptosis, and melanogenesis in different types of skin residential cells. The different biological effects of those EMW therapies have been applied in treatment of several human diseases. Clinically, several diseases are treated with UVB-based phototherapy, including psoriasis, atopic dermatitis, vitiligo, cutaneous T-cell lymphomas, and morphea [98] because UVB have an effect on cell proliferation, apoptosis, and immunomodulation. Due to the development of modern UVB lamps that are easily applicable, UVB phototherapy is more often used than PUVA. At the present moment, PUVA is very seldom used in the USA and Europe. However, even though UVB phototherapy is very efficient in the treatment of psoriasis, it is less frequently used by dermatologist than it was 10–20 years ago [106,107]. Narrowband UVB has increased efficacy in psoriasis treatment over broadband UVB and is safer than PUVA [108].

UVA1 can be used to treat many diseases and has been shown scientifically to be effective in the treatment of localized scleroderma and atopic dermatitis. UVA1 induces cyclobutane pyrimidine dimers but not 6–4 photoproducts in human skin *in vivo*[109]. UVA1 exerts its therapeutic effects through T cell apoptosis, collagenase induction, angiogenesis, tissue remodeling [97]. Psoralen ultraviolet A (PUVA), on the other hand, is a form of chemophototherapy which utilizes UVA to activate psoralens, a photoreactive chemical [110]. When irradiated with UVA, psoralens can inhibit DNA replication and cause cell cycle arrest and ultimately apoptosis. Psoralen photosensitization also causes an alteration in the expression of cytokines and cytokine receptors [111]. Psoralens directly interact with RNA, proteins and other cellular components and indirectly modify them via ROS [112]. Epidermal and dermal infiltrating lymphocytes are robustly suppressed by PUVA, with varying effects on different T-cell subsets [113]. Like UVB, PUVA can also stimulate melanogenesis and inhibit immune responses [114].

Photodynamic therapy (PDT) utilizes an exogenous photosensitizer that is preferentially absorbed by tumor cells, endothelial cells, and active inflammatory cells. Once the cells harboring the photosensitizer are irradiated with light fitting the absorption spectrum, the target cells are destroyed by production of ROS and execution of the apoptotic cascade. PDT is used clinically to treat a wide range of medical conditions, including light-accessible premalignant and malignant cancers [104]. The most widely used photosenstizer is ALA, which can convert to porphyrins in the tissue [115]. Due to its potent effect in inducing lymphocyte apoptosis, extra-corporeal photopheresis is used to treat erythrodermic cutaneous lymphomas. Despite the introduction of several effective biological agents in medicine and dermatology, phototherapy remains a reliable and preferred option for treatment of several dermatological diseases.

## 9. Clinical Implications of UV-Induced Apoptosis

### 9.1. Keratinocytes

In several inflammatory skin diseases, or so-called “interface or lichenoid dermatitis,” inflammatory skin cells line up in the superficial dermis and attack the overlying epidermis. These lichenoid reactions are found in several diseases, including lichen planus, chronic graft-versus-host disease, lichenoid drug reaction, Stevens Johnson syndrome, and lupus erythematosus (LE). These inflammatory cells cause scattered keratinocyte apoptosis, which manifests itself as dyskeratotic cells and/or a cytoid body and as a positive deposition in direct immuno-fluorescent assay (DIF). Notably, patients with LE have the common feature of photosensitivity. Abnormal generation and clearance of UV-induced apoptotic keratinocytes in LE are an important source of autoantigens. UV induces apoptosis, resulting in the generation of chemokines, which recruit more effector memory T cells and plasmacytoid DCs in UV-induced cutaneous LEs lesions [116]. A second clinical example featuring UV-induced apoptosis is the response of psoriasis to UV phototherapy. UV-induced keratinocyte apoptosis occurs after irradiation of psoriatic plaque *in vivo.* The degree of keratinocyte apoptosis may be a useful biomarker indicating clinical response to different UV spectra [117]. A third example is in the effect of keratinocyte apoptosis in the development of arsenic-induced skin cancers. Both UV and arsenic induces skin cancers. UV-induced skin cancers tend to occur solitarily in sun-exposed skin whereas arsenic-induced skin cancers tend to occur in multiplicity and are distributed in sun-spared skin. The fact that arsenic-induced skin cancers do not usually occur in sun-exposed skin might result from the synergistic apoptotic effects of UV and arsenic in keratinocytes, in which the combined use of UVB irradiation and arsenic treatment results in the pro-apoptotic effects induced by activation of Caspase 8 and Caspase 9 [44,45].

### 9.2. Melanocytes

Melanins, produced by melanocytes, play an important role in protecting the skin against UV radiation. Skin cancers occur more often in individuals with light skin. UV-induced DNA damage in melanocytes is more effectively prevented in darker skin due to an enhanced UV-induced apoptosis. The decrease of DNA damage with more efficient removal of UV-damaged cells may contribute at least in part to the decreased prevalence of skin cancers in individuals with dark skin [118]. On the other hand, once the melanocytes escape the apoptosis check, they can undergo malignant transformation to one of the most fatal cancers in the human, malignant melanoma [119]. In therapeutical applications, the narrow band UVB induce repigmentation in vitiligo by inhibition of self-destruction against external stresses [120] and by promotion of melanocyte regeneration [121,122].

### 9.3. Skin-Associated Lymphoid Tissues

It is well known that patients in chronic immunosuppression status (such as patients with organ transplants receiving intense immunosuppressive therapy to prevent rejection) are prone to develop skin cancers, primarily in areas exposed to the sun [123]. Thus, an intact immune system is important in the immune surveillance and clearance of UV-induced apoptotic cells in carcinogenesis of the UV-induced skin cancers. This is reflected in currently available murine xenograft tumor models, which show that most of UV-induced cancers fail to grow when transplanted to normal, syngeneic mice but grow progressively in immunosuppressed mice [124]. The defects in immune surveillance might result from the generation of tolerogenic LCs or dermal dendritic cells [125] by UV-induced apoptotic cells, with subsequent activation of Treg cells and depletion of CD8^+^ T cells [65]. With regard to their relative therapeutic effects, the therapeutic effects in patients with psoriasis, both UVB and PUVA equally reduce lymphocytes, macrophages and dendritic cells in psoriatic skin. However, only PUVA can decrease the epidermal LCs [126]. After UV irradiation, the loss of epidermal LCs results from apoptosis and/or defective migration to draining LNs [127,128]. In mast cells, UVR radiation and topical 1,25(OH)_2_D3 can also activate dermal mast cells [88,89], which are important determinants of the extent of UV immunomodulation [89,90]. UVA1 radiation induces collagenase (matrix metalloproteinase-1) expression, T-cell apoptosis, and depletes Langerhans and mast cells in the dermis. UVA1 exposure stimulates neovascularization in endothelial cells [129]. The absorption of UV in endothelial cells preferentially uptaking porphyrins might help explain the therapeutic base for photodynamic therapy. In peripheral sensory nerves, UVR enhances stimulation of sensory nerves and increases production of neuropeptides, prompting recall immunological response [130].

## 10. Conclusions

UV radiation is absorbed by nuclear DNA, initiating a cascade of apoptotic events by forming DNA photoproducts and suppressing DNA synthesis. UV radiation can also cause damage to the molecular targets located in the cytosol and cell membranes of keratinocytes. UV may result in the depletion of epidermal LCs and dermal T lymphocytes, which then lead to Treg activation in the lymph nodes. The articles reviewed in this study suggest that DNA damage, induction of apoptosis, immune suppression, alteration in cytokine profiles, and induction of cell signaling pathways may contribute to the effects of UV-based phototherapy. Today different UVB sources, narrowband UVB lamps, a 308-nm UVB excimer laser, UVA or UVA1 lamps are widely applied for treatment of inflammatory, sclerosing, and neoplastic conditions including atopic dermatitis, sclerosing skin conditions such as morphea, vitiligo, and mycosis fungoides. Understanding the detailed mechanisms through which phototherapy using different UV sources exert its effect as well as a better understanding of the pathophysiology of various diseases should help open up the paths for future therapeutic options.

## 11. Perspectives

Phototherapy has more than 100 years of history. In the era of targeting therapy, it remains the mainstay of treatment for many cutaneous inflammatory diseases, simply because many of these diseases are not caused by the defect of a single gene/protein that could be targeted by biologics. The many mechanistic aspects and physiological nature of the phototherapy may contribute to its advantage in the treatment or prevention of diseases without the use of pharmaceutical drugs. In addition, different phototherapies using different wavelengths and different combinations have different biological effects on cell differentiation, tissue remodeling, tissue regeneration, apoptosis, and neovascularization. The precondition by light other than UV may prevent the harmful effects of UV in theory, which is the basic concept of photoprevention analogue to the concept of chemoprevention, which prevents the occurrence and progression of neoplasia. The health benefit of photorejuvenization relies on the biological effects of light on the remodeling of tissues. The concept of photoregeneration in the skin might be applied in future phototherapy in the regeneration of other vital cells such as neurons and myocardial cells. On the other hand, the possible chronic health effects of light-emitting diodes (LEDs) and other newly-introduced light devices as light sources in our daily life or as a therapeutic modality remain to be investigated.

## Figures and Tables

**Table 1:** Application of phototherapies in the treatment of diseases and the mechanisms of actions.

Light source	Targets	Mechanisms	Diseases
***1. Ablation***			
CO_2_ laser (10,800 nm) Er-YAG laser (3850 nm)	Water in and outside the cells	Evaporation	Superficial skin tumors [94]

***2. Non-ablation***			
Dye laser	Hemoglobin	Photoselective thermolysis	Vascular lesions: Hemangioma and telangiectasia [95]

Ruby laser (694 nm) Alexander laser (700–820 nm)	Melanin	Photoselective thermolysis	Pigmentary lesions: Lentigenes Freckles melanocytic nevi [96]

***3. UV***			
UVA1 (340–400 nm)	Chormophores	T cell apoptosis Collagenase induction Angiogenesis Tissue remodeling	Atopic dermatitis Localized scleroderma [97]

UVB			
(Broadband and narrowband) (Excimer light/lasers)	Chromophores	Anti-inflammation, Melanogenesis Apoptosis	Psoriasis vitiligo Atopic dermatitis Intractable pruritus [98]

***4. Chemophototherapy***			
PUVA (Psoralen + UVA)	Psoralen, DNA	ROS production DNA replication inhibition Cell cycle arrest Apoptosis Anti-inflammation Melanogenesis	Cutaneous T cell lymphoma Skin mastocytosis [99,100]

***5. Low power lasers/light/LEDs***			
IR or visible light	Chromophores	Immunomodulation Tissue remodeling Melanogenesis Analgesic	Vitiligo Chronic wound Neuralgia [101,102]

Blue-Green visible light	Bilirubin	Photoisomerization Photodegradation	Neonatal jaundice [92]

***6. Other Phototherapies***			
Extra-corporeal photopheresis	Chromophore	T cells depletion	Erythrodermic cutaneous T cell lymphoma [103]

Photodynamic therapy	Photosensitizers	ROS production Apoptosis	Superficial skin cancer [104]

## References

[1] Swann G. (2010). The skin is the body’s largest organ. J. Vis. Commun. Med.

[2] Grice E.A., Segre J.A. (2011). The skin microbiome. Nat. Rev. Microbiol.

[3] Madison K.C. (2003). Barrier function of the skin: “La raison d’etre” of the epidermis. J. Invest. Dermatol.

[4] Pincelli C., Marconi A. (2010). Keratinocyte stem cells: Friends and foes. J. Cell Physiol.

[5] Gray-Schopfer V., Wellbrock C., Marais R. (2007). Melanoma biology and new targeted therapy. Nature.

[6] Koch S., Kohl K., Klein E., von Bubnoff D., Bieber T. (2006). Skin homing of Langerhans cell precursors: Adhesion, chemotaxis, and migration. J. Allergy Clin. Immunol.

[7] Bennett C.L., Noordegraaf M., Martina C.A., Clausen B.E. (2007). Langerhans cells are required for efficient presentation of topically applied hapten to T cells. J. Immunol.

[8] Lugo L.M., Lei P., Andreadis S.T. (2011). Vascularization of the dermal support enhances wound re-epithelialization by in situ delivery of epidermal keratinocytes. Tissue Eng. Part A.

[9] Hayward M.G., Keatinge W.R. (1981). Roles of subcutaneous fat and thermoregulatory reflexes in determining ability to stabilize body temperature in water. J. Physiol.

[10] Diffey B.L. (2002). Sources and measurement of ultraviolet radiation. Methods.

[11] Krutmann J. (2001). The role of UVA rays in skin aging. Eur. J. Dermatol.

[12] Coelho S.G., Hearing V.J. (2010). UVA tanning is involved in the increased incidence of skin cancers in fair-skinned young women. Pigment Cell Melanoma Res.

[13] Krutmann J. (2000). Ultraviolet A radiation-induced biological effects in human skin: Relevance for photoaging and photodermatosis. J. Dermatol. Sci.

[14] Phan T.A., Halliday G.M., Barnetson R.S., Damian D.L. (2006). Spectral and dose dependence of ultraviolet radiation-induced immunosuppression. Front. Biosci.

[15] Batista L.F., Kaina B., Meneghini R., Menck C.F. (2009). How DNA lesions are turned into powerful killing structures: Insights from UV-induced apoptosis. Mutat. Res.

[16] Setlow R.B., Carrier W.L. (1966). Pyrimidine dimers in ultraviolet-irradiated DNA’s. J. Mol. Biol.

[17] D’Errico M., Lemma T., Calcagnile A., de Santis L.P., Dogliotti E. (2007). Cell type and DNA damage specific response of human skin cells to environmental agents. Mutat. Res.

[18] Pfeifer G.P. (1997). Formation and processing of UV photoproducts: Effects of DNA sequence and chromatin environment. Photochem. Photobiol.

[19] Ziegler A., Jonason A.S., Leffell D.J., Simon J.A., Sharma H.W., Kimmelman J., Remington L., Jacks T., Brash D.E. (1994). Sunburn and p53 in the onset of skin cancer. Nature.

[20] Pierceall W.E., Kripke M.L., Ananthaswamy H.N. (1992). N-*ras* mutation in ultraviolet radiation-induced murine skin cancers. Cancer Res.

[21] Jiveskog S., Ragnarsson-Olding B., Platz A., Ringborg U. (1998). N-*ras* mutations are common in melanomas from sun-exposed skin of humans but rare in mucosal membranes or unexposed skin. J. Invest. Dermatol.

[22] Kulms D., Schwarz T. (2000). Molecular mechanisms of UV-induced apoptosis. Photodermatol. Photoimmunol. Photomed.

[23] Assefa Z., van Laethem A., Garmyn M., Agostinis P. (2005). Ultraviolet radiation-induced apoptosis in keratinocytes: On the role of cytosolic factors. Biochim. Biophys. Acta.

[24] Green E.A., Choi Y., Flavell R.A. (2002). Pancreatic lymph node-derived CD4(+)CD25(+) Treg cells: Highly potent regulators of diabetes that require TRANCE-RANK signals. Immunity.

[25] Takasawa R., Nakamura H., Mori T., Tanuma S. (2005). Differential apoptotic pathways in human keratinocyte HaCaT cells exposed to UVB and UVC. Apoptosis.

[26] Mouret S., Philippe C., Gracia-Chantegrel J., Banyasz A., Karpati S., Markovitsi D., Douki T. (2010). UVA-induced cyclobutane pyrimidine dimers in DNA: A direct photochemical mechanism?. Org. Biomol. Chem.

[27] Courdavault S., Baudouin C., Charveron M., Canguilhem B., Favier A., Cadet J., Douki T. (2005). Repair of the three main types of bipyrimidine DNA photoproducts in human keratinocytes exposed to UVB and UVA radiations. DNA Repair.

[28] Greinert R., Volkmer B., Henning S., Breitbart E.W., Greulich K.O., Cardoso M.C., Rapp A. (2012). UVA-induced DNA double-strand breaks result from the repair of clustered oxidative DNA damages. Nucleic Acids Res.

[29] Zhan Q., Fan S., Smith M.L., Bae I., Yu K., Alamo I., O’Connor P.M., Fornace A.J. (1996). Abrogation of p53 function affects gadd gene responses to DNA base-damaging agents and starvation. DNA Cell Biol..

[30] Hoeijmakers J.H. (2009). DNA damage, aging, and cancer. N. Engl J. Med.

[31] Schoppy D.W., Ruzankina Y., Brown E.J. (2010). Removing all obstacles: A critical role for p53 in promoting tissue renewal. Cell Cycle.

[32] Tron V.A., Trotter M.J., Tang L., Krajewska M., Reed J.C., Ho V.C., Li G. (1998). p53-regulated apoptosis is differentiation dependent in ultraviolet B-irradiated mouse keratinocytes. Am. J. Pathol.

[33] Rodust P.M., Stockfleth E., Ulrich C., Leverkus M., Eberle J. (2009). UV-induced squamous cell carcinoma—A role for antiapoptotic signalling pathways. Br. J. Dermatol.

[34] Bang B., Gniadecki R., Larsen J.K., Baadsgaard O., Skov L. (2003). *In vivo* UVB irradiation induces clustering of Fas (CD95) on human epidermal cells. Exp. Dermatol.

[35] Aragane Y., Kulms D., Metze D., Wilkes G., Poppelmann B., Luger T.A., Schwarz T. (1998). Ultraviolet light induces apoptosis via direct activation of CD95 (Fas/APO-1) independently of its ligand CD95L. J. Cell Biol.

[36] Tobin D., van Hogerlinden M., Toftgard R. (1998). UVB-induced association of tumor necrosis factor (TNF) receptor 1/TNF receptor-associated factor-2 mediates activation of Rel proteins. Proc. Natl. Acad. Sci. USA.

[37] Qin J.Z., Bacon P.E., Chaturvedi V., Bonish B., Nickoloff B.J. (2002). Pathways involved in proliferating, senescent and immortalized keratinocyte cell death mediated by two different TRAIL preparations. Exp. Dermatol.

[38] Qin J.Z., Bacon P., Panella J., Sitailo L.A., Denning M.F., Nickoloff B.J. (2004). Low-dose UV-radiation sensitizes keratinocytes to TRAIL-induced apoptosis. J. Cell Physiol.

[39] Kroemer G. (1997). The proto-oncogene Bcl-2 and its role in regulating apoptosis. Nat. Med.

[40] Riedl S.J., Salvesen G.S. (2007). The apoptosome: Signalling platform of cell death. Nat. Rev. Mol. Cell Biol.

[41] Assefa Z., Garmyn M., Vantieghem A., Declercq W., Vandenabeele P., Vandenheede J.R., Agostinis P. (2003). Ultraviolet B radiation-induced apoptosis in human keratinocytes: Cytosolic activation of procaspase-8 and the role of Bcl-2. FEBS Lett.

[42] Van Laethem A., van Kelst S., Lippens S., Declercq W., Vandenabeele P., Janssens S., Vandenheede J.R., Garmyn M., Agostinis P. (2004). Activation of p38 MAPK is required for Bax translocation to mitochondria, cytochrome c release and apoptosis induced by UVB irradiation in human keratinocytes. FASEB J.

[43] Lee C.H., Yu C.L., Liao W.T., Kao Y.H., Chai C.Y., Chen G.S., Yu H.S. (2004). Effects and interactions of low doses of arsenic and UVB on keratinocyte apoptosis. Chem. Res. Toxicol.

[44] Lee C.H., Liao W.T., Yu H.S. (2011). Aberrant immune responses in arsenical skin cancers. Kaohsiung J. Med. Sci.

[45] Yu H.S., Liao W.T., Chai C.Y. (2006). Arsenic carcinogenesis in the skin. J. Biomed. Sci.

[46] Holley A.K., St. Clair D.K. (2009). Watching the watcher: Regulation of p53 by mitochondria. Future Oncol.

[47] Wu Y., Xing D., Liu L., Gao B. (2008). Regulation of Bax activation and apoptotic response to UV irradiation by p53 transcription-dependent and -independent pathways. Cancer Lett.

[48] Moysan A., Clement-Lacroix P., Michel L., Dubertret L., Morliere P. (1996). Effects of ultraviolet A and antioxidant defense in cultured fibroblasts and keratinocytes. Photodermatol. Photoimmunol. Photomed.

[49] Podda M., Traber M.G., Weber C., Yan L.J., Packer L. (1998). UV-irradiation depletes antioxidants and causes oxidative damage in a model of human skin. Free Radic Biol. Med.

[50] Halliday G.M. (2005). Inflammation, gene mutation and photoimmunosuppression in response to UVR-induced oxidative damage contributes to photocarcinogenesis. Mutat. Res.

[51] Birch-Machin M.A., Swalwell H. (2010). How mitochondria record the effects of UV exposure and oxidative stress using human skin as a model tissue. Mutagenesis.

[52] Lai W.W., Hsiao Y.P., Chung J.G., Wei Y.H., Cheng Y.W., Yang J.H. (2011). Synergistic phototoxic effects of glycolic acid in a human keratinocyte cell line (HaCaT). J. Dermatol. Sci.

[53] Liu C.Y., Lee C.F., Wei Y.H. (2007). Quantitative effect of 4977 bp deletion of mitochondrial DNA on the susceptibility of human cells to UV-induced apoptosis. Mitochondrion.

[54] Liu C.Y., Lee C.F., Wei Y.H. (2009). Role of reactive oxygen species-elicited apoptosis in the pathophysiology of mitochondrial and neurodegenerative diseases associated with mitochondrial DNA mutations. J. Formos Med. Assoc.

[55] Paz M.L., Gonzalez Maglio D.H., Weill F.S., Bustamante J., Leoni J. (2008). Mitochondrial dysfunction and cellular stress progression after ultraviolet B irradiation in human keratinocytes. Photodermatol. Photoimmunol. Photomed.

[56] Schroeder P., Gremmel T., Berneburg M., Krutmann J. (2008). Partial depletion of mitochondrial DNA from human skin fibroblasts induces a gene expression profile reminiscent of photoaged skin. J. Invest. Dermatol.

[57] Timares L., Katiyar S.K., Elmets C.A. (2008). DNA damage, apoptosis and langerhans cells—Activators of UV-induced immune tolerance. Photochem. Photobiol.

[58] Beissert S., Ruhlemann D., Mohammad T., Grabbe S., El-Ghorr A., Norval M., Morrison H., Granstein R.D., Schwarz T. (2001). IL-12 prevents the inhibitory effects of *cis*-urocanic acid on tumor antigen presentation by Langerhans cells: Implications for photocarcinogenesis. J. Immunol.

[59] Sreevidya C.S., Fukunaga A., Khaskhely N.M., Masaki T., Ono R., Nishigori C., Ullrich S.E. (2010). Agents that reverse UV-Induced immune suppression and photocarcinogenesis affect DNA repair. J. Invest. Dermatol.

[60] Hart P.H., Gorman S., Finlay-Jones J.J. (2011). Modulation of the immune system by UV radiation: More than just the effects of vitamin D?. Nat. Rev. Immunol.

[61] Voll R.E., Herrmann M., Roth E.A., Stach C., Kalden J.R., Girkontaite I. (1997). Immunosuppressive effects of apoptotic cells. Nature.

[62] Kolgen W., Both H., van Weelden H., Guikers K.L., Bruijnzeel-Koomen C.A., Knol E.F., van Vloten W.A., de Gruijl F.R. (2002). Epidermal langerhans cell depletion after artificial ultraviolet B irradiation of human skin *in vivo*: Apoptosis versus migration. J. Invest. Dermatol.

[63] Nishigori C., Yarosh D., O’Connor A., Shreedhar V.K., Ullrich S.E., Cox P., Kripke M.L. (1998). HindIII liposomes suppress delayed-type hypersensitivity responses *in vivo* and induce epidermal IL-10 *in vitro*. J. Immunol.

[64] Kripke M.L., Cox P.A., Bucana C., Vink A.A., Alas L., Yarosh D.B. (1996). Role of DNA damage in local suppression of contact hypersensitivity in mice by UV radiation. Exp. Dermatol.

[65] Loser K., Mehling A., Loeser S., Apelt J., Kuhn A., Grabbe S., Schwarz T., Penninger J.M., Beissert S. (2006). Epidermal RANKL controls regulatory T-cell numbers via activation of dendritic cells. Nat. Med.

[66] Elmets C.A., Bergstresser P.R., Tigelaar R.E., Wood P.J., Streilein J.W. (1983). Analysis of the mechanism of unresponsiveness produced by haptens painted on skin exposed to low dose ultraviolet radiation. J. Exp. Med.

[67] Chen A., Xu H., Choi Y., Wang B., Zheng G. (2004). TRANCE counteracts FasL-mediated apoptosis of murine bone marrow-derived dendritic cells. Cell Immunol.

[68] Cremer I., Dieu-Nosjean M.C., Marechal S., Dezutter-Dambuyant C., Goddard S., Adams D., Winter N., Menetrier-Caux C., Sautes-Fridman C., Fridman W.H. (2002). Long-lived immature dendritic cells mediated by TRANCE-RANK interaction. Blood.

[69] Josien R., Li H.L., Ingulli E., Sarma S., Wong B.R., Vologodskaia M., Steinman R.M., Choi Y. (2000). TRANCE, a tumor necrosis factor family member, enhances the longevity and adjuvant properties of dendritic cells *in vivo*. J. Exp. Med.

[70] Dudda J.C., Denfeld R.W., Simon J.C., Martin S.F. (2004). UVB-irradiated dendritic cells fail to tolerize murine CD8 naive or effector T cells. J. Invest. Dermatol.

[71] Garssen J., Vandebriel R.J., de Gruijl F.R., Wolvers D.A., van Dijk M., Fluitman A., van Loveren H. (1999). UVB exposure-induced systemic modulation of Th1- and Th2-mediated immune responses. Immunology.

[72] Johnson-Huang L.M., Suarez-Farinas M., Sullivan-Whalen M., Gilleaudeau P., Krueger J.G., Lowes M.A. (2010). Effective narrow-band UVB radiation therapy suppresses the IL-23/IL-17 axis in normalized psoriasis plaques. J. Invest. Dermatol.

[73] Walters I.B., Ozawa M., Cardinale I., Gilleaudeau P., Trepicchio W.L., Bliss J., Krueger J.G. (2003). Narrowband (312-nm) UV-B suppresses interferon gamma and interleukin (IL) 12 and increases IL-4 transcripts: Differential regulation of cytokines at the single-cell level. Arch. Dermatol.

[74] Pradhan S., Kim H.K., Thrash C.J., Cox M.A., Mantena S.K., Wu J.H., Athar M., Katiyar S.K., Elmets C.A., Timares L. (2008). A critical role for the proapoptotic protein bid in ultraviolet-induced immune suppression and cutaneous apoptosis. J. Immunol.

[75] Kobayashi N., Nakagawa A., Muramatsu T., Yamashina Y., Shirai T., Hashimoto M.W., Ishigaki Y., Ohnishi T., Mori T. (1998). Supranuclear melanin caps reduce ultraviolet induced DNA photoproducts in human epidermis. J. Invest. Dermatol.

[76] Abdel-Malek Z.A., Kadekaro A.L., Swope V.B. (2010). Stepping up melanocytes to the challenge of UV exposure. Pigment. Cell Melanoma Res.

[77] Smith A.G., Luk N., Newton R.A., Roberts D.W., Sturm R.A., Muscat G.E. (2008). Melanocortin-1 receptor signaling markedly induces the expression of the NR4A nuclear receptor subgroup in melanocytic cells. J. Biol. Chem.

[78] Song X., Mosby N., Yang J., Xu A., Abdel-Malek Z., Kadekaro A.L. (2009). alpha-MSH activates immediate defense responses to UV-induced oxidative stress in human melanocytes. Pigment. Cell Melanoma Res.

[79] Ravnbak M.H., Wulf H.C. (2007). Pigmentation after single and multiple UV-exposures depending on UV-spectrum. Arch. Dermatol. Res.

[80] Miller S.A., Coelho S.G., Zmudzka B.Z., Bushar H.F., Yamaguchi Y., Hearing V.J., Beer J.Z. (2008). Dynamics of pigmentation induction by repeated ultraviolet exposures: Dose, dose interval and ultraviolet spectrum dependence. Br. J. Dermatol.

[81] MacLaughlin J.A., Anderson R.R., Holick M.F. (1982). Spectral character of sunlight modulates photosynthesis of previtamin D3 and its photoisomers in human skin. Science.

[82] Nesby-O’Dell S., Scanlon K.S., Cogswell M.E., Gillespie C., Hollis B.W., Looker A.C., Allen C., Doughertly C., Gunter E.W., Bowman B.A. (2002). Hypovitaminosis D prevalence and determinants among African American and white women of reproductive age: Third National Health and Nutrition Examination Survey, 1988–1994. Am. J. Clin. Nutr.

[83] Garland C.F., Garland F.C., Gorham E.D., Lipkin M., Newmark H., Mohr S.B., Holick M.F. (2006). The role of vitamin D in cancer prevention. Am. J. Public Health.

[84] Hewison M. (2010). Vitamin D and the intracrinology of innate immunity. Mol. Cell Endocrinol.

[85] Ellison T.I., Smith M.K., Gilliam A.C., MacDonald P.N. (2008). Inactivation of the vitamin D receptor enhances susceptibility of murine skin to UV-induced tumorigenesis. J. Invest. Dermatol.

[86] Di Rosa M., Malaguarnera M., Nicoletti F., Malaguarnera L. (2011). Vitamin D3: A helpful immuno-modulator. Immunology.

[87] Liu P.T., Stenger S., Li H., Wenzel L., Tan B.H., Krutzik S.R., Ochoa M.T., Schauber J., Wu K., Meinken C. (2006). Toll-like receptor triggering of a vitamin D-mediated human antimicrobial response. Science.

[88] Biggs L., Yu C., Fedoric B., Lopez A.F., Galli S.J., Grimbaldeston M.A. (2010). Evidence that vitamin D(3) promotes mast cell-dependent reduction of chronic UVB-induced skin pathology in mice. J. Exp. Med.

[89] Hart P.H., Grimbaldeston M.A., Swift G.J., Jaksic A., Noonan F.P., Finlay-Jones J.J. (1998). Dermal mast cells determine susceptibility to ultraviolet B-induced systemic suppression of contact hypersensitivity responses in mice. J. Exp. Med.

[90] Hart P.H., Townley S.L., Grimbaldeston M.A., Khalil Z., Finlay-Jones J.J. (2002). Mast cells, neuropeptides, histamine, and prostaglandins in UV-induced systemic immunosuppression. Methods.

[91] (1904). Obituary: Niels Ryberg Finsen, M.D. Brit. Med. J..

[92] Moller K.I., Kongshoj B., Philipsen P.A., Thomsen V.O., Wulf H.C. (2005). How Finsen’s light cured lupus vulgaris. Photodermatol. Photoimmunol. Photomed.

[93] Martineau A.R., Honecker F.U., Wilkinson R.J., Griffiths C.J. (2007). Vitamin D in the treatment of pulmonary tuberculosis. J. Steroid Biochem. Mol. Biol.

[94] Maisels M.J., McDonagh A.F. (2008). Phototherapy for neonatal jaundice. N. Engl. J. Med.

[95] Roll E.B., Christensen T. (2005). Formation of photoproducts and cytotoxicity of bilirubin irradiated with turquoise and blue phototherapy light. Acta Paediatr.

[96] Tierney E.P., Hanke C.W., Petersen J. (2012). Ablative fractionated CO_2_ laser treatment of photoaging: A clinical and histologic study. Dermatol. Surg.

[97] Hunzeker C.M., Geronemus R.G. (2010). Treatment of superficial infantile hemangiomas of the eyelid using the 595-nm pulsed dye laser. Dermatol. Surg.

[98] Raulin C., Schonermark M.P., Greve B., Werner S. (1998). Q-switched ruby laser treatment of tattoos and benign pigmented skin lesions: A critical review. Ann. Plast. Surg.

[99] Breuckmann F., Appelhans C., Bastian A., Stuecker M., Altmeyer P., Kreuter A. (2004). UVA1-induced decrease in dermal neuron-specific enolase (NSE) in acrosclerosis. Arch. Dermatol. Res.

[100] Stern R.S. (2007). Psoralen and ultraviolet a light therapy for psoriasis. N. Engl. J. Med.

[101] Stadler R. (2007). Optimal combination with PUVA: Rationale and clinical trial update. Oncology.

[102] Wolff K. (2002). Treatment of cutaneous mastocytosis. Int. Arch. Allergy Immunol.

[103] Hu W.P., Wang J.J., Yu C.L., Lan C.C., Chen G.S., Yu H.S. (2007). Helium-neon laser irradiation stimulates cell proliferation through photostimulatory effects in mitochondria. J. Invest. Dermatol.

[104] Lan C.C., Wu C.S., Chiou M.H., Hsieh P.C., Yu H.S. (2006). Low-energy helium-neon laser induces locomotion of the immature melanoblasts and promotes melanogenesis of the more differentiated melanoblasts: Recapitulation of vitiligo repigmentation *in vitro*. J. Invest. Dermatol.

[105] Atta M., Papanicolaou N., Tsirigotis P. (2012). The role of extracorporeal photopheresis in the treatment of cutaneous T-cell lymphomas. Transfus. Apher. Sci.

[106] Lee Y., Baron E.D. (2011). Photodynamic therapy: Current evidence and applications in dermatology. Semin. Cutan. Med. Surg.

[107] Bulat V., Situm M., Dediol I., Ljubicic I., Bradic L. (2011). The mechanisms of action of phototherapy in the treatment of the most common dermatoses. Coll. Antropol.

[108] Housman T.S., Rohrback J.M., Fleischer A.B., Feldman S.R. (2002). Phototherapy utilization for psoriasis is declining in the United States. J. Am. Acad. Dermatol..

[109] Wan J., Abuabara K., Troxel A.B., Shin D.B., van Voorhees A.S., Bebo B.F., Krueger G.G., Callis Duffin K., Gelfand J.M. (2012). Dermatologist preferences for first-line therapy of moderate to severe psoriasis in healthy adult patients. J. Am. Acad. Dermatol..

[110] Lapolla W., Yentzer B.A., Bagel J., Halvorson C.R., Feldman S.R. (2011). A review of phototherapy protocols for psoriasis treatment. J. Am. Acad. Dermatol.

[111] Tewari A., Sarkany R.P., Young A.R. (2012). UVA1 induces cyclobutane pyrimidine dimers but not 6-4 photoproducts in human skin *in vivo*. J. Invest. Dermatol.

[112] Epstein J.H. (1990). Phototherapy and photochemotherapy. N. Engl. J. Med.

[113] Mermelstein F.H., Abidi T.F., Laskin J.D. (1989). Inhibition of epidermal growth factor receptor tyrosine kinase activity in A431 human epidermoid cells following psoralen/ultraviolet light treatment. Mol. Pharmacol.

[114] Averbeck D. (1989). Recent advances in psoralen phototoxicity mechanism. Photochem. Photobiol.

[115] Volkmar C.M., Vukadinovic-Walter B., Oplander C., Bozkurt A., Korth H.G., Kirsch M., Mahotka C., Pallua N., Suschek C.V. (2010). UVA-induced phenoxyl radical formation: A new cytotoxic principle in photodynamic therapy. Free Radic Biol. Med.

[116] Lehmann P., Homey B. (2009). Clinic and pathophysiology of photosensitivity in lupus erythematosus. Autoimmun. Rev.

[117] Weatherhead S.C., Farr P.M., Reynolds N.J. (2013). Spectral effects of UV on psoriasis. Photochem. Photobiol. Sci.

[118] Yamaguchi Y., Takahashi K., Zmudzka B.Z., Kornhauser A., Miller S.A., Tadokoro T., Berens W., Beer J.Z., Hearing V.J. (2006). Human skin responses to UV radiation: Pigment in the upper epidermis protects against DNA damage in the lower epidermis and facilitates apoptosis. FASEB J.

[119] Bivik C.A., Larsson P.K., Kagedal K.M., Rosdahl I.K., Ollinger K.M. (2006). UVA/B-induced apoptosis in human melanocytes involves translocation of cathepsins and Bcl-2 family members. J. Invest. Dermatol.

[120] Yoshiki R., Nakamura M., Tokura Y. (2012). The biological role of UVB-induced cutaneous immunosuppression. J. UOEH.

[121] Dong D., Jiang M., Xu X., Guan M., Wu J., Chen Q., Xiang L. (2012). The effects of NB-UVB on the hair follicle-derived neural crest stem cells differentiating into melanocyte lineage *in vitro*. J. Dermatol. Sci.

[122] Wu C.S., Yu C.L., Lan C.C., Yu H.S. (2004). Narrow-band ultraviolet-B stimulates proliferation and migration of cultured melanocytes. Exp. Dermatol.

[123] Euvrard S., Kanitakis J., Claudy A. (2003). Skin cancers after organ transplantation. N. Engl. J. Med.

[124] Kripke M.L. (2013). Reflections on the field of photoimmunology. J. Invest. Dermatol.

[125] Wang L., Jameson S.C., Hogquist K.A. (2009). Epidermal Langerhans cells are not required for UV-induced immunosuppression. J. Immunol.

[126] Erkin G., Ugur Y., Gurer C.K., Asan E., Korkusuz P., Sahin S., Kolemen F. (2007). Effect of PUVA, narrow-band UVB and cyclosporin on inflammatory cells of the psoriatic plaque. J. Cutan. Pathol.

[127] Hamakawa M., Sugihara A., Okamoto H., Horio T. (2006). Ultraviolet B radiation suppresses Langerhans cell migration in the dermis by down-regulation of alpha4 integrin. Photodermatol. Photoimmunol. Photomed.

[128] Tang A., Udey M.C. (1992). Effects of ultraviolet radiation on murine epidermal Langerhans cells: Doses of ultraviolet radiation that modulate ICAM-1 (CD54) expression and inhibit Langerhans cell function cause delayed cytotoxicity *in vitro*. J. Invest. Dermatol.

[129] Zandi S., Kalia S., Lui H. (2012). UVA1 phototherapy: A concise and practical review. Skin Therapy Lett.

[130] Howes R.A., Halliday G.M., Barnetson R.S., Friedmann A.C., Damian D.L. (2006). Topical capsaicin reduces ultraviolet radiation-induced suppression of Mantoux reactions in humans. J. Dermatol. Sci.

